# CHD1L prevents lipopolysaccharide-induced hepatocellular carcinomar cell death by activating hnRNP A2/B1-nmMYLK axis

**DOI:** 10.1038/s41419-021-04167-9

**Published:** 2021-09-29

**Authors:** Guangliang Wang, Xiaofeng Zhang, Wei Cheng, Yanxuan Mo, Juan Chen, Zhiming Cao, Xiaogang Chen, Huiqin Cui, Shanshan Liu, Li Huang, Ming Liu, Lei Ma, Ning-Fang Ma

**Affiliations:** 1 Affiliated Cancer Hospital and Institute, Guangzhou Medical University, Guangzhou, China; 2 Department of Histology and Embryology, Faculty of Basic Medical Sciences, Guilin Medical University, Guilin, Guangxi China; 3 Guangzhou Municipal and Guangdong Provincial Key Laboratory of Protein Modification and Degradation, School of Basic Medical Sciences, Guangzhou Medical University, Guangzhou, China

**Keywords:** Cancer microenvironment, Apoptosis, Liver cancer

## Abstract

Chromodomain helicase/ATPase DNA-binding protein 1-like gene (CHD1L) has been characterized to be a driver gene in hepatocellular carcinoma (HCC). However, the intrinsic connections between CHD1L and intestinal dysbacteriosis-related inflammation reaction in HCC progression remain incompletely understood. In this study, a specific correlation between CHD1L and nonmuscle isoform of myosin light chain kinase (nmMLCK/nmMYLK), a newly identified molecule associated NF-κB signaling transduction, was disclosed in HCC. CHD1L promotes nmMYLK expression and prevents lipopolysaccharide (LPS) induced tumor cell death. In vitro experiment demonstrated that overexpressed nmMYLK is essential for CHD1L to maintain HCC cell alive, while knocking down nmMYLK significantly attenuate the oncogenic roles of CHD1L. Mechanism analysis revealed that nmMYLK can prevent Caspase-8 from combining with MyD88, an important linker of TLRs signaling pathway, while, knocking down nmMYLK facilitate the MyD88 combines with Caspase-8 and lead to the proteolytic cascade of Caspase as well as the consequent cell apoptosis. Mechanism analysis showed that CHD1L promotes the nmMYLK expression potentially through upregulating the heterogeneous nuclear ribonucleoproteins A2/B1 (hnRNP A2/B1) expression, which can bind to myosin light chain kinase (*MYLK*) pre-mRNA and lead to the regnant translation of nmMYLK. In summary, this work characterizes a previously unknown role of CHD1L in preventing LPS-induced tumor cell death through activating hnRNP A2/B1-nmMYLK axis. Further inhibition of CHD1L and its downstream signaling could be a novel promising strategy in HCC treatment.

## Introduction

Dysbacteriosis, characterized by excessive lipopolysaccharide-induced endotoxemia and activation of TLRs-NF-κB signaling, might occur after antibiotic therapy, surgery, chemotherapy, or radiotherapy of cancer. Increasing evidence suggested that dysbacteriosis was associated with the progression of tumor. Chromodomain helicase/adenosine triphosphatase DNA-binding protein 1-like gene (*CHD1L*) is originally verified in human hepatocellular carcinoma cells (HCC) and its oncogenic roles have been characterized in previous studies [1, 2]. Many studies revealed the oncogenic roles of CHD1L in different kinds of malignancies, such as ovarian carcinoma [3], bladder cancer [4], colorectal carcinoma [5], breast cancer [6], and lung adenocarcinoma [7], etc. Considering that dysbacteriosis is common in cancer patients, it is important to know how CHD1L works in the pathological microenvironment of dysregulated flora.

Myosin light chain kinase (MLCK/MYLK) has been complicated in tumor cell proliferation, invasion, and distant metastasis [8–10]. MYLK gene encodes mainly two different catalytic protein isoforms. The short isoform named smooth muscle MYLK (smMYLK) is mainly expressed in smooth muscle tissue, while the long isoform generally exists in nonmuscle tissues (nmMYLK) [11]. Recent study shows that nmMYLK involves in NF-κB-dependent transcription of inflammation related genes in endothelial cells [12]. Our unpublished high-throughput sequencing data indicated that nmMYLK was closely correlated with CHD1L expression in HCC, which hints a potential relationship between CHD1L and NF-κB signaling transduction.

In this study, the correlations between CHD1L and nmMYLK and their roles to tumor cells under LPS mimetic inflammatory condition were investigated, and nmMYLK was found to play an important role in CHD1L-promoted tumor cell proliferation and antiapoptotic effects. CHD1L upregulates heterogeneous nuclear ribonucleoproteins A2/B1 (hnRNP A2/B1) expression, which binding to MYLK pre-mRNA and leads to the regnant translation of nmMYLK. These findings suggested that CHD1L-regulated hnRNP A2/B1/nmMYLK axis was important in preventing LPS-induced tumor cell death. Blocking this critical axis might be a promising strategy in HCC treatment.

## Results

### CHD1L promotes nmMYLK expression in HCC

**Fig. 1:**
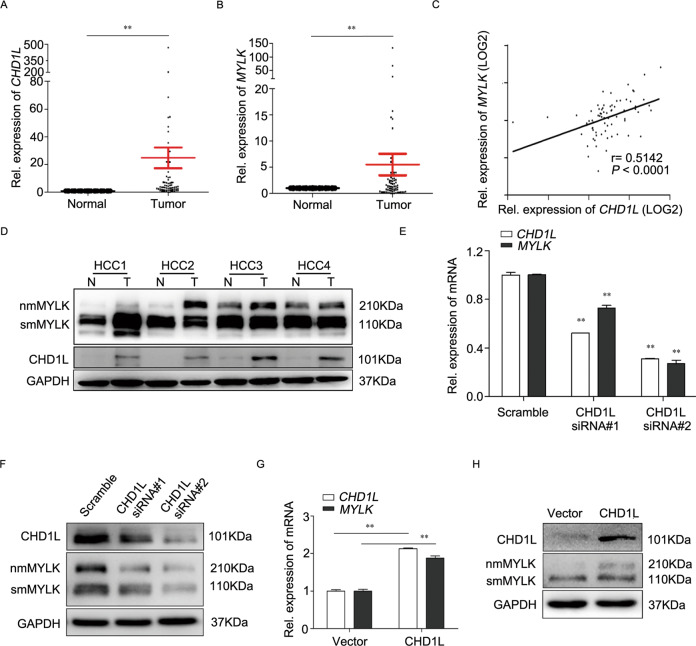
CHD1L promotes nmMYLK expression in HCC. **A, B** Quantitative RT-PCR (qRT-PCR) analysis for *CHD1L* and *MYLK* in 73 pairs of HCC tumors (T) and adjacent nontumor tissues (N). Data shown as mean ± SD. **C** Spearman correlation analysis for the expression of CHD1L and MYLK (r = 0.5142, *P* < 0.0001), based on qRT-PCR results. **D** Western blot assay was used to detect the expression of CHD1L and MYLK in paired HCC samples, with GAPDH as normalized control. **E, F** The mRNA and protein levels of CHD1L and MYLK in HepG2 cells were detected with qRT-PCR and western blot after siCHD1L treatment, Data were performed by mean with SD from three independent experiments. **G, H** The mRNA and protein levels of CHD1L and MYLK in 7703 cells were detected using qRT-PCR and western blot. Data were performed by mean with SD from three independent experiments. **P < 0.01.

To further study the mechanism of CHD1L, we have constructed the CHD1L overexpressed HCC cell line. QGY-7703 cells were transfected with CHD1L-GFP or Vector plasmid, the total RNA of CHD1L-GFP or Vector stable cell lines were extracted and checked for high-throughput sequencing. By analyzing the throughput mRNA sequencing database, we identified MYLK as a potential downstream gene of CHD1L (unpublished data). To confirm the relationship between CHD1L and MYLK, the endogenous expression of CHD1L and MYLK in different HCC cell lines including LO2, QGY-7703, HepG2, Huh7 was measured by qRT-PCR and western blot (Supplementary Fig. S1). Similar trends in *CHD1L* and *MYLK* expression were detected from qRT-PCR analysis (Supplementary information, Fig. S1A). Western blot analysis indicated smMYLK expression levels in all tested cell lines were similar; however, an additional nmMYLK band was appeared in HepG2, Huh7 cells, the cells possess a high level expression of CHD1L (Supplementary information, Fig. S1B), indicated the nmMYLK, rather than smMYLK, was tightly correlated with CHD1L expression. As a result, we further examined the correlation between CHD1L and nmMYLK in 73 paired HCC specimens. Overexpression of CHD1L and nmMYLK was observed in 64/73 (87%) and 43/73 (59%) HCC samples, when compared with the para-nontumorous counterparts (Fig. 1A, B). A positive correlation was shown using Spearman’s correlation analysis (87% vs. 59%, Spearman correlation coefficient, 0.5142; *P* < 0.0001) (Fig. 1C). The correlation between MYLK expression and clinicopathological features of 73 HCC patients was evaluated. High MYLK expression was positively correlated with HBsAg (*P* = 0.04), microvascular invasion (*P* = 0.031), and tumor encapsulation (*P* = 0.028) (Table 1). Western blot results from some of the paired HCC specimens showed that the expression of nmMYLK was particularly prominent in the tumor specimens with CHD1L overexpression, as compared with the basal level of smMYLK (Fig. 1D). To further confirm this relationship, CHD1L-specific small-interfering RNA (siRNA) was used to imitate CHD1L downregulation in HepG2. And the expression of nmMYLK was substantially reduced corresponding to the downregulation of CHD1L as compared with the nonspecific siRNA control (Fig. 1E, F).

**Table 1:** Association of MYLK expression with clinicopathological characteristics in 73 HCC samples.

Clinicopathological features	*n*	MYLK expression		High MYLK expression (%)	χ^2^ value	*P* value
		High (*n* = 43)	Low (*n* = 30)			
Sex						
Female	9	2	7	22	5.706	0.017
Male	64	41	23	64		
Age (years)						
≤60	57	31	26	54	2.193	0.139
>60	16	12	4	75		
Serum AFP (ng/ml)						
≤252	34	24	10	71	3.589	0.058
>252	39	19	20	49		
HBsAg						
Negative	16	13	3	81	4.227	0.04
Positive	57	30	27	53		
Liver cirrhosis						
Absence	12	8	4	67	0.357	0.55
Presence	61	35	26	57		
Tumor number						
Single	47	24	23	51	3.351	0.067
Multiple	26	19	7	73		
Maximal tumor size (cm)						
≤3	9	5	4	56	0.048	0.827
>3	64	38	26	59		
Barcelona staging						
0,A and B	49	28	21	57	0.191	0.662
C and D	24	15	9	63		
Tumor encapsulation						
Absence	28	12	16	43	4.832	0.028
Presence	45	31	14	69		
Microvascular invasion						
Absence	10	9	1	90	4.629	0.031
Presence	63	34	29	54		
Metastasis						
Absence	71	43	30	58	1.435	0.231
Presence	2	2	0	100		

An alternative approach was applied by transfecting 7703 cells with CHD1L expression plasmid. Upregulation of CHD1L increased the expression of MYLK in 7703 cells. Similar to that Supplementary Fig. S1B, MYLK isoforms were expressed differently in responding to the CHD1L upregulation. Besides the basal expression of smMYLK, nmMYLK was specifically expressed in 7703-CHD1L cells, as compared with its vector control (Fig. 1G, H). These results suggest a positive regulatory role of CHD1L on nmMYLK expression.

### The essential role of nmMYLK in promoting the proliferation and antiapoptosis of HCC cells

**Fig. 2:**
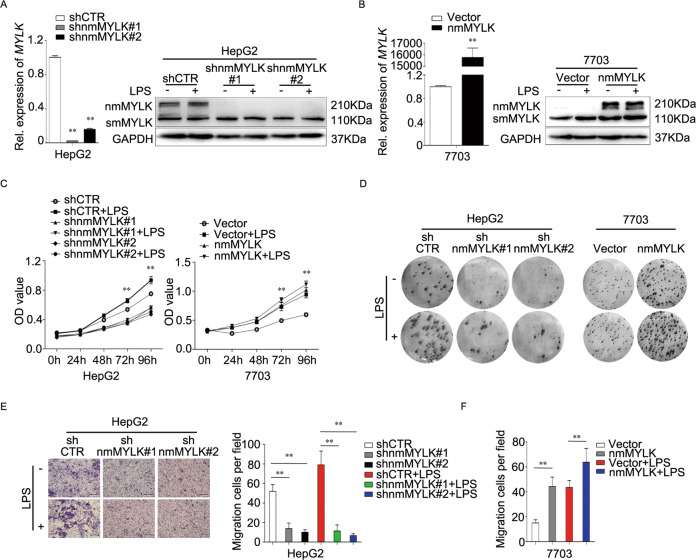
nmMYLK promote the proliferation of HCC cells. **A–F** HepG2 cells were transfected with shRNA-nmMYLK lentivirus or lentivirus-vector control before LPS treatment, 7703 cells were transfected with nmMYLK expression constructs or its vector before LPS treatment. The expressions of MYLK in HepG2 and 7703 cells were detected using qRT-PCR and western blot (**A, B**). Cell proliferation abilities were examined using MTS assay (**C**) and Colony formation assay (**D**). Cell migration abilities were examined using Transwell test (**E, F**). The experiments were repeated three times independently, and the bars represent SD. **P < 0.01.

Considering the special anatomical environment of the liver and the involvements of nmMYLK in NF-κB-dependent transcription of inflammatory or survival related genes, we applied exogenous LPS to the culture system to mimic the inflammatory environment. To assess the general effect of nmMYLK on malignant phenotype of HCC cells and to understand its mechanism, we transduced HepG2 with shRNAs targeting *nmMYLK* (hereafter referred to as sh*nmMYLK*) or a non-targeting shRNA (hereafter referred to as shCTR). An alternative approach was applied by transfecting 7703 cells with *nmMYLK* expression plasmid. Accordingly, the expression of *nmMYLK* was determined by using qRT-PCR and Western blotting. And down or upregulation of nmMYLK in HepG2 or 7703 cells was significantly more efficient than their vector controls (Fig. 2A, B). MTS analysis was applied to evaluate the effects of nmMYLK on HCC cells proliferation. The results indicated that downregulation of nmMYLK inhibited HepG2 cell proliferation with or without LPS treatment (Fig. 2C, Left). On the contrary, the enforced expression of nmMYLK increased tumor cells proliferation with or without LPS treatment (Fig. 2C, Right). Similar results were observed in the colony formation assay with that of MTS assay (Fig. 2D).

Downregulation of nmMYLK induced potent suppression on HepG2 cells migration. After treated with LPS, the migration cells/field in both the shCTR and nmMYLK downregulated cells (shnmMYLK) were increased, but the latter was lower than that of the former (Fig. 2E). Overexpressed nmMYLK in 7703 cells significantly reversed this phenotype when compared with the vector control cells, and this effect was enhanced after LPS treatment (Fig. 2F).

**Fig. 3:**
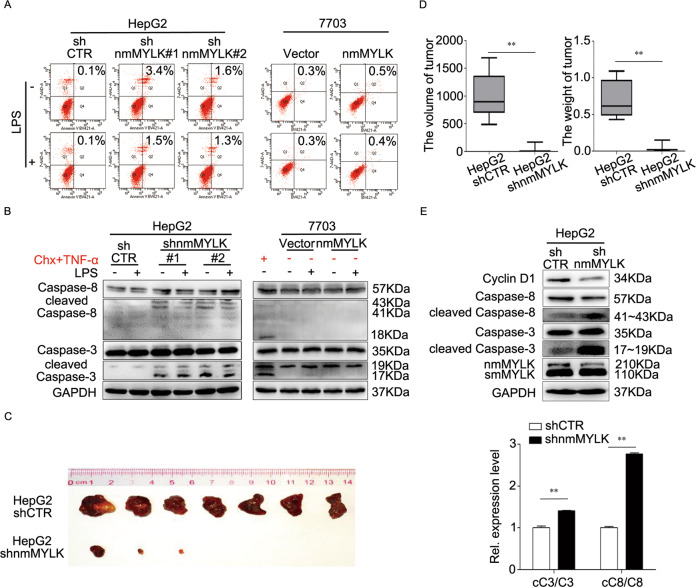
Downregulation of nmMYLK in HCC cells promotes tumor cells apoptosis and attenuates tumor formation in nude mice. **A, B** HepG2 cells were transfected with shRNA-nmMYLK lentivirus or lentivirus-vector control before LPS treatment, 7703 cells were transfected with nmMYLK expression constructs or its vector before LPS treatment. Flow cytometry (**A**) and western blot (**B**) were used for examining the apoptosis rate of HepG2 and 7703 cells. **C, D** Mice xenograft models were constructed by inoculated HepG2 cells subcutaneously. Tumors dissected from different groups (**C**), tumor volumes and weight analysis (**D**). **E** Relative cascade reactions of caspases in xenograft tumors were examined using western blot. Data were performed by mean with SD from three independent experiments. **P < 0.01.

To explore the molecular mechanism involved in nmMYLK mediated tumor cell survival, the effects of nmMYLK on apoptosis were further evaluated. Flow Cytometry assays revealed that downregulated nmMYLK could enhance apoptosis. The apoptotic indexes were 0.1% and 3.4% or 1.6% in HepG2-shCTR cells and HepG2-shnmMYLK cells, respectively. LPS reduced the apoptosis of tumor cells, but the apoptosis rate reminded higher in HepG2-shnmMYLK cells than that of control (Fig. 3A, Left). No significant difference was observed in the apoptotic index of 7703-nmMYLK and 7703-vector cells with and without LPS treatment (Fig. 3A, Right).

The expression levels of cleaved Caspase-9, Caspase-8, Caspase-3, and Cyclin D1 in aforesaid cell lines was detected and the results showed that the cleaved Caspase-3/8 was increased in HepG2-shnmMYLK cells and Cyclin D1 experienced a greater reduction than that of control cells when treated with or without LPS (Fig. 3B, Left and Supplementary Fig. S2A). No significant apoptosis was found in 7703 Vector or nmMYLK cells, which mean the lower basal apoptosis level, and weak cleaved Caspase-3/8 bends were only detected after Chx + TNF-α treatment. And the expression of Cyclin D1 was clearly upregulated in nmMYLK overexpressed 7703 cells as compared with its vector control (Fig. 3B, Right and Supplementary Fig. S2B).

The tumorigenic effect of nmMYLK was verified in nude mice through subcutaneous implantation of HepG2-shnmMYLK and its control HepG2-shCTR cells. The size and weight of the tumor from the shnmMYLK-transfected HepG2 cells were smaller than that of control (990.56 ± 401.38 mm^3^ vs 61.67 ± 94.69 mm^3^, *P* < 0.01; 0.69 ± 0.25 g vs 0.06 ± 0.08 g, *P* < 0.01) (Fig. 3C, D). The expression of Cyclin D1 reduced and cleaved Caspase-3/8 increased obviously in HepG2-shnmMYLK group, comparing with the control group (Fig. 3E). To summarize, the results from gain- or loss-of-function studies indicate that nmMYLK mediated tumor cell proliferation and apoptosis.

### nmMYLK is essential for CHD1L-promoted malignant proliferation in tumor cells

**Fig. 4:**
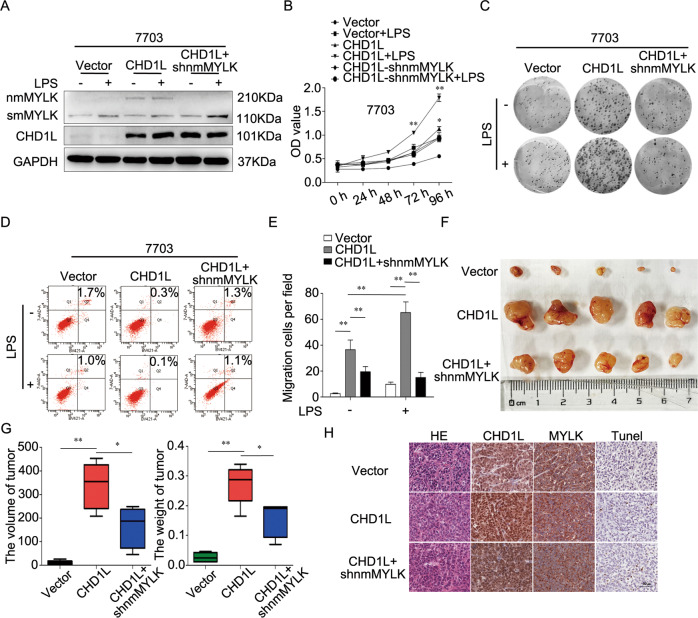
nmMYLK is essential for CHD1L-promoted malignant proliferation in tumor cells. **A**–**H** 7703 overexpression CHD1L cells were infected with specific shRNA (shnmMYLK) lentivirus. Western blot was applied to detect the expression of CHD1L and MYLK (**A**). Cell proliferation ability was examined using MTS assay (**B**) and Colony formation assay (**C**). Cell apoptosis level was examined using flow cytometry analysis (**D**). Cell migration ability was examined using Transwell test (**E**). 7703-CHD1L cells infected with shnmMYLK lentiviruses and inoculated subcutaneously into nude mice, following by monitoring for tumor growth, Tumor pictures (**F**), Tumor volumes and weight (**G**). Tumor tissues were embedded with paraffin and subjected to H&E and immunohistochemistry analysis (20×), Scale bar = 50 μm, representative images were presented (**H**). Data were performed by mean with SD from three independent experiments. *P < 0.05, **P < 0.01.

To clarify the importance of nmMYLK for CHD1L to promote the malignant proliferation of HCC by infecting 7703-CHD1L cells with shnmMYLK lentivirus (refered as 7703-CHD1L-shnmMYLK), the expressions of nmMYLK and CHD1L were evaluated (Fig. 4A). MTS assay showed that LPS promoted the proliferation of 7703-CHD1L cells and its vector control, and downregulation of nmMYLK inhibited CHD1L-induced cell proliferation. LPS could not rescue the effects of the downregulation of nmMYLK on cell proliferation (Fig. 4B). The clone formation assay got the same conclusion (Fig. 4C). Flow Cytometry assay revealed a lower apoptotic index from CHD1L overexpressed cells than that of vector control (0.3% *vs* 1.7%). while, knockdown nmMYLK increased the apoptosis indexes in 7703-CHD1L cells (0.5% up to 1.3%) (Fig. 4D). Transwell assays showed that downregulation of nmMYLK in 7703-CHD1L cells significantly decreased cell migration, which could not be rescued by an additional treatment of LPS when compared with 7703-CHD1L cells (Fig. 4E). To confirm the in vivo effects of nmMYLK on CHD1L-promoted tumor growth, 7703-Vector, 7703-CHD1L, and 7703-CHD1L-shnmMYLK cell line were constructed and applied for subcutaneous implantation in nude mice. As shown in Fig. 4F, G, knockdown nmMYLK attenuated the tumorigenic capacity of CHD1L. Tunnel and western blot analysis showed increased apoptosis rates in 7703-CHD1L-shnmMYLK implantation group (Fig. 4H and Supplementary Fig. S3). These results indicated that nmMYLK is essential for CHD1L-promoted malignant growth.

### CHD1L-mediated nmMYLK expression was depended on hnRNP A2/B1

**Fig. 5:**
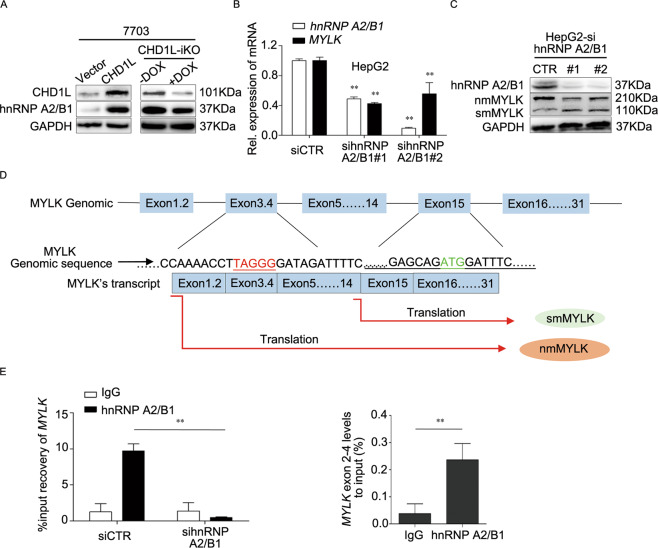
CHD1L controlled nmMYLK expression depends on hnRNP A2/B1. **A** Protein levels of hnRNP A2/B1 and CHD1L were evaluated in 7703-CHD1L cells and 7703-CHD1L-iKO cells. **B, C** qRT-PCR and western blot were used to detect the expression levels of hnRNP A2/B1 and MYLK in HepG2 cells after treated with sihnRNP A2/B1 or scramble small-interfering RNA, **P < 0.01. **D** Schematic depiction of hnRNP A2/B1 binding sites on *MYLK*. **E** The binding ability of hnRNP A2/B1 to MYLK’s pre-mRNA was examined by Immunoprecipitation (RIP) assay in HepG2-sihnRNP A2/B1 cells or scramble control. **P < 0.01.

HnRNPs are a class of multifunctional proteins belonging to the heterogeneous ribonuclear protein family, and involve in processing the heterogeneous nuclear RNA into messenger RNA [13, 14], we’ve accidentally found that the heterogeneous nuclear ribonucleoprotein A2/B1 (hnRNP A2/B1) was upregulated in CHD1L overexpression cells (Fig. 5A). A core component being hnRNP A2/B1 that is involved in alternative splicing [15, 16], and it was reported that hnRNP A1 is involved in the alternate splicing of MYLK [17]. The effect of hnRNP A2/B1 on MYLK splicing was validated by transfecting HepG2 cells with hnRNP A2/B1-specific siRNA, Western blot indicated that knockdown hnRNP A2/B1 could inhibit the expression of nmMYLK, while the expression of smMYLK was upregulated (Fig. 5B, C). Previous studies showed that hnRNP A2/B1 specifically binds to UAGGG sequences [18, 19]. We analyzed the full-length DNA and protein sequences of MYLK, and found there is a TAGGG motif in the exon 3–4 and ATG in the exon 15 of *MYLK* with ~2800 bps length. MYLK protein domain analysis showed that nmMYLK possess a specialized N-terminal domain containing 922-aa, which cover the motif ranged from exon 1 to exon 15, contains more 2766 bps of nucleic acid sequence than that of smMYLK (Fig. 5D). RNA Immunoprecipitation (RIP) experiment further verified that hnRNP A2/B1 indeed binds to the pre-mRNA of *MYLK*, more exactly, its binding site was at the exon 3–4 region (Fig. 5E). The above results indicated that CHD1L promotes nmMYLK formation via upregulating hnRNP A2/B1, which can bind to the pre-mRNA of MYLK and protect it from splicing.

### nmMYLK is essential for LPS-induced TLR4/NF-κB signal transduction

**Fig. 6:**
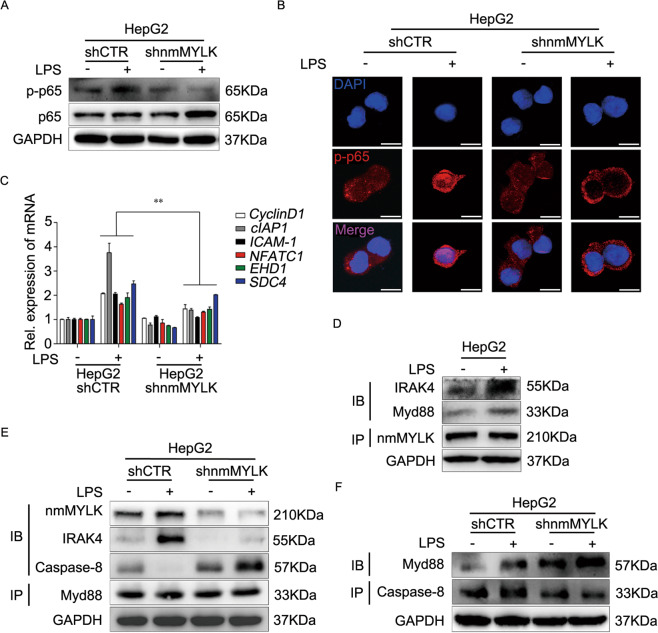
nmMYLK is needed for LPS-induced NF-κB activation and apoptosis inhibition. **A, B** The expression and localization of p65 and p-p65 were detected by western blot and IF assays in HepG2 cells with or without LPS treatment. Scale bar = 10 μm. **C** The mRNA levels of Relative NF-κB DTGs were examined in HepG2 cells with or without LPS treatment. **D** The co-IP assay was carried out in HepG2 cells with or without LPS treatment for verifying the interaction between Myd88, IRAK4, and nmMYLK. **E** Related binding capacities of Myd88 to nmMYLK, IRAK4, and Caspase-8 were examined by co-IP assay in HepG2 cells after shRNA transfection with or without LPS treatment. **F** The co-IP assay was carried out in HepG2-shCTR and HepG2 cells after shRNA transfection with or without LPS treatment for verifying the interaction capacity of Caspase-8 to Myd88, nmMYLK, and IRAK4. Data were shown as mean ± SD from three independent experiments. **P < 0.01.

nmMYLK was involved in NF-κB-dependent inflammatory signal transduction and promoted the transcription of surviving genes in vascular endothelial cells [20, 21]. We next investigated whether the same effects of nmMYLK were existed in tumor cells. Previous study has shown that the LPS induces Ca^2+^ entry in a TLR4-dependent manner and the internal flow of Ca^2+^ could activate nmMYLK [12]. In this regard, we applied Fura-4-AM to determine cytosolic Ca^2+^ concentration in responding to LPS. And found that LPS caused an increased intracellular Ca^2+^ concentration in HepG2 cells (Supplementary Fig. S4A). To validate LPS induces nmMYLK enzymatic activity, we observed that phosphorylated MLC2 was detected after LPS treatment, which representing the activation of nmMYLK (Supplementary Fig. S4B). Considering the aforementioned effects of LPS on nmMYLK in tumor cells, we speculated that, as the receptor of LPS, TLR4, and its downstream NF-κB signaling pathway should be involved. Then, the activity of the key subunit of NF-κB, the phosphorylated p65 and its downstream target genes (DTGs) expression in HepG2-shnmMYLK cells were detected. Western blot and immunofluorescence assay indicated that LPS induced p65 phosphorylation and nuclear translocation (Fig. 6A, B). No significant differences in the nmMYLK downregulated cells were found (Fig. 6A, B). LPS increased the transcription of NF-κB DTGs, such as *Cyclin D1*, *cIAP1*, *ICAM-1*, *NFATC1*, *EHD1*, and *SDC4* in HepG2 cells had been reported previously [22]. Herein, as compared to HepG2-shCTR cells, only a slight increase was observed in HepG2-shnmMYLK cells (Fig. 6C), indicating that nmMYLK is essential for LPS-induced NF-κB activation.

**Fig. 7:**
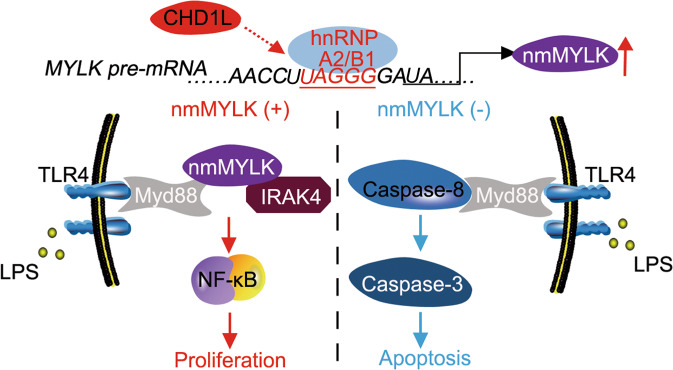
Schematic diagram depicts a proposed model for the regulation and mechanism of nmMYLK in HCC. CHD1L upregulates hnRNP A2/B1 to promise the nmMYLK formation. Abundant nmMYLK might interact with MyD88 and IRAK4 to activate NF-κB and its downstream signaling, which finally promoting the proliferation of tumor cells. The Depleted nmMYLK enable MyD88 to interact with Caspase-8 and lead to the cascade activation reaction of caspase proteins, which finally result in tumor cell apoptosis.

To ascertain the possible link between nmMYLK related NF-κB activation and MyD88-IRAK4 complex, lysates from LPS treated HepG2 cells were immunoprecipitated with anti-nmMYLK antibody, followed by immunoblotting with anti-MyD88 and anti-IRAK4 antibody to evaluate their interactions. The results found that nmMYLK could combine with MyD88 and IRAK4, which was strengthened after LPS treatment (Fig. 6D). Likewise, using anti-MyD88 antibody, unexpected results showed that besides nmMYLK and IRAK4, the apoptotic protein Caspase-8 also interacted with MyD88 and oppositely correlated with nmMYLK abundance, and knockdown nmMYLK enabled MyD88 to interact with Caspase-8 (Fig. 6E). To confirm whether MyD88 interacts with Caspase-8, anti-Caspase-8 antibody was used to repeat co-immunoprecipitation. Similar results were obtained, which indicated a real binding between Caspase-8 and MyD88. However, no results showed a possible binding between Caspase-8 and nmMYLK or IRAK4. Downregulation of nmMYLK increased the interaction of Caspase-8 with MyD88 (Fig. 6F). The above results indicated a new mechanism of CHD1L in preventing LPS-induced tumor cell death via activating hnRNP A2/B1-nmMYLK axis (Fig. 7).

## Discussion

As one of SNF2 superfamily member, CHD1L possessing putative helicase sequence motifs is similar to those found in helicase superfamily 2, which confers the ability for stabilizing or perturbing protein-DNA interaction [23]. CHD1L protein contains two helicase domains, a C-terminal macro domain and a nuclear localization sequence, which function as a transcription regulator to affect a broad spectrum of cellular processes [24]. CHD1L regulates downstream genes transcription by directly binding to the promoters of related target gene, such as *ARHGEF9*, *TCTP*, *Nur77*, and *SPOCK1* to maintain HCC cell survival, migration, invasion, and inhibits apoptosis [25–28]. Here, we demonstrate that the nmMYLK expression is closely depending on CHD1L in HCC. Overexpression of nmMYLK was detected in over 59% of HCC cases with a positive correlation with CHD1L expression.

MYLK and its spliced isoforms play important roles in regulating cell survival, proliferation, and motility [29, 30]. MYLK has been reported to phosphorylate myosin light chains (MLC) and regulate the invasion and metastasis of HCC and some other malignancies, which were mainly focused on MYLK subtype smMYLK (108–130 kD) [31]. In this study, the results from in vitro and in vivo validation proved that nmMYLK, not smMYLK, is essential in CHD1L driven HCC cancer malignant phenotype, noticeable, all of these effects partially depended on LPS mimicked dysbacteriosis situation.

Previous studies demonstrated that nmMYLK was relates to NF-κB-dependent inflammatory and the transcription of survival genes in acute lung injury [21, 32]. Herein, we confirmed that nmMYLK promotes HCC cell proliferation by activating NF-κB signaling and upregulating its downstream genes *Cyclin D1*, *cIAP1*, *ICAM-1*, *NFATC1*, *EHD1*, and *SDC4* that are correlated with the malignant phenotype of some kinds of tumors. This indicated that nmMYLK plays a specific role in LPS-induced malignancies.

Apoptosis inhibition is associated with tumor development. Caspase-8, executor of apoptosis, influences cell adhesion and migration through post-cleavage remodeling of cytoskeleton and signaling elements that control focal adhesion turnover [33]. Our study showed that knocking down nmMYLK increased the accumulation of cytoplasmic Caspase-8 and trigger the proteolytic cascade reaction. However, the same effects were not observed in nmMYLK overexpressed 7703 cells. It might be due to the suppression of apoptosis-related genes in tumor cells. Accumulation of cleaved Caspases-8 and Caspases-3 induced by downregulated nmMYLK led to cell death, in addition, the oncogenic effects of CHD1L on HCC cells were significantly blocked, suggesting nmMYLK was critical for CHD1L. Both the gain- or loss-of function tests supported that the CHD1L associated oncogenic role of nmMYLK was closely correlated with Caspase-8 inactivation.

Heterogeneous nuclear ribonucleoproteins (hnRNPs) plays an essential role in regulating gene expression, including RNA splicing, polyadenylation, etc [34, 35]. hnRNP A2/B1 was implicated in the progress of breast cancer, head and neck cancer, non-small cell lung cancer, and prostate cancer [15, 36–38]. Recent studies indicated hnRNP A2/B1 could interact with miR-503HG to control the metastasis of HCC and NF-κB signaling pathway [39]. Here, a positive correlation between CHD1L and hnRNP A2/B1 was identified. Specifically, CHD1L upregulated the expression of hnRNP A2/B1 in HCC cells. Meanwhile, knocking down hnRNP A2/B1 could reduce the expression ratio of nmMYLK/smMYLK. RIP experiments confirmed that hnRNP A2/B1 could bind to the motif of the MYLK pre-mRNA and lead to the long transcript of nmMYLK formation in HCC cells. Besides, there are two smMYLK and nmMYLK bands were observed in HepG2 and Huh7 cells in Fig. S1B. The possible reason is due to the high internal level of CHD1L, which upregulated hnRNP A2/B1 expression and subsequent control the alternate splicing of MYLK. Given these, we speculate that the two bands of smMYLK and nmMYLK probably belong to their different isoforms. Because hnRNP A2/B1 dominantly promotes the long isoform nmMYLK formation, logically, the cells that express high level CHD1L would generate more nmMYLK, and vice the versa. With the same reason, we can interpret that why the expression levels of smMYLK were lower in HepG2 and Huh7 cells than that in LO2 and 7703 cells, which is because the former expressed higher level of CHD1L than the latter one does.

In view of LPS-related reactions and the oncogenic roles of nmMYLK in HCC, the mechanism of nmMYLK for transmitting LPS-TLR4 signaling to its downstream NF-κB pathway was undisclosed. It was evident that TLR4 delivered immune responses through activating its adaptor MyD88 and recruiting IRAK4, which then phosphorylates IRAK1 and IRAK2 to induce NF-κB activity [40]. Besides its role in mediating actomyosin, nmMYLK could also function as a scaffold mediating protein to promote protein interactions via its 922-aa N-terminal domain [41]. Consistent with the studies in endothelial cells [12], nmMYLK could interact with MyD88 and IRAK4 in HCC cells, this phenomenal was further strengthened by LPS treatment. Interestingly, MyD88 failed to bind IRAK4 in response to LPS in nmMYLK downregulated cells. These observations best fit a model that LPS may induce Ca^2+^ influx and activate nmMYLK consequently, which could function as a scaffold to support the interaction of MyD88-IRAK4, and finally promote the transcription of NF-κB downstream genes. It is known that the oligomerization of MyD88 death domain was critical for the interaction of MyD88 and IRAK4 [42]. Although nmMYLK has no canonical death domain, the serine/threonine residues in MyD88 domain might serve as the substrates of nmMYLK, which results in the oligomerization of MyD88 and its interaction with IRAK4 [42]. Even so, the above reports were still insufficient to explain the effects of downregulated nmMYLK on cell apoptosis.

TLRs signal transduction through two main pathway: one is MyD88 combined with IRAK1/4 to activate NF-κB pathway, the other is MyD88 interacted with Fas-associated with death domain protein (FADD) and transmit signaling to Caspase-8 to induce apoptosis [43]. It can be deducted that downregulated nmMYLK might prevent MyD88 from binding to IRAK4, which gives the chance to MyD88 to integrate with FADD-Caspase-8. Lots of studies have proven that MyD88 could interacts with other proteins, although the mechanisms were not known completely [44]. Our study provides a possibility that the Caspase-8 may bind to MyD88. Caspase-8 contains two death effector domains (DED). When the ligands exist, the inhibitor of death domain on the receptor’s intracellular peptide will dissociate and expose the intracellular peptide DD domains [45]. MyD88 also contains a DD domain and can offer a structural basis for interacting with Caspase-8, which has been recently proved to be involved in cell cytoskeletal remodeling and migration in a number of cell types [46]. Our study shows the role of nmMYLK in the interaction of MyD88 with IRAK4 or Caspase-8. We speculate that MyD88 could bind to Caspase-8 and trigger the cascade of Caspase, which in turn activates downstream apoptotic signaling molecules, initiates cell apoptosis and inhibits cell migration. This mode clearly explains the oncogenic roles of nmMYLK in HCC cells.

Taken together, our results suggested that nmMYLK is a critical effector of CHD1L in response to LPS-induced TLR4/MyD88 signaling, which depends on the phenotype of HCC cells differently. Overexpression of nmMYLK worked synergistically with CHD1L to promote HCC cells proliferation. While, downregulation of nmMYLK blocked the malignant phenotype of HCC cells induced by LPS/TLR4/MyD88/NF-κB signaling, promoted MyD88 binding with Caspase-8 to trigger cell apoptosis.

In summary, the results above suggest that nmMYLK functions as a key molecule for CHD1L to respond to LPS/TLR4/MyD88/NF-κB signaling. And abolishment of CHD1L and nmMYLK might block the promotion of LPS on malignant cells proliferation, which might be a novel therapeutic strategy for HCC treatment.

## Materials and methods

### Cell lines, tumor specimens

LO2, QGY-7703, Huh7, and HepG2 cell lines were applied and their background information has been described in previous studies [28]. Cells were cultured in DMEM supplemented with 10% fetal bovine serum. LPS was purchased from Sigma–Aldrich (L2880) and 1 μg/ml LPS was used to treat HCC cells in this study. HCC tissues used were reviewed and approved by the Committees for Ethical Review of Research involving Human Subjects of Guangzhou Medical University and Sun Yat-Sen University. A total of 73 paired human HCC and adjacent nontumor (NT) tissues were obtained from patients with informed consent for the use of their clinical specimens for medical research.

### Animals

Four-week-old pathogen-free male Balb/c-nu mice were obtained from Guangzhou University of Chinese Medicine Experimental Animal Center. Mice were housed in the pathogen-free laboratory animal center at Guangzhou Medical University. All animal experiments were performed in accordance with the Institutional Animal Care and Use Committee protocols of Guangzhou Medical University.

### Antibodies

Anti-human CHD1L (ab51324), CHD1L (ab197019), anti-MYLK (ab232949), anti-Cyclin D1 (ab134175), and anti-hnRNP A2/B1 (ab31645) antibodies were purchased from Abcam. Anti-human GAPDH (#5174), anti-NF-κB pathway sampler kit (#9936), Apoptosis Antibody Sampler Kit (#9930), anti–rabbit IgG (#6990), anti–MLC2 sampler kit (#9776), anti-MyD88 (#4283), and anti-IRAK4 (#4363) Proteins were purchased from Cell Signaling Technology (CST).

### Vector construction and small-interfering RNA transfection

Cells were cultivated to 50–60% confluence and were then treated with siRNA or shRNA (sequences of used siRNA and shRNA was listed in Table S1) according to the manufacturer’s instructions. For overexpression of *nmMYLK* (MYLK-GFP plasmid presented by Addgene), cells were harvested in 48 h after transfection. For MYLK knockdown assay, green fluorescent protein (GFP)-tagged shMYLK was cloned into pLenti6/V5-TOPO vector (Life Technologies) and the viruses were packaged and transfected into HepG2 cells according to the manufacturer’s instructions.

### MTS assay

Cells were seeded in 96-well plates and cultured. After treated with the indicated conditions, the MTS solution (Promega, USA) was added into each well and incubated at 37 °C for 2 h. The optical density (OD) at 490 nm were measured by a microplate reader at different time periods according to the manufacturer’s protocol.

### Migration assay

Transwell chamber (8 μm, 24-well format; FALCON) was inserted into the culture plates. Cells in 0.2 mL serum-free mediums were seeded in the upper chamber. The migrated cells in transwell chambers were fixed with methanol for 20 min after culturing, stained with crystal violet and counted in five random fields under the microscope.

### Colony formation assay

Cells were cultured in six-well plate with indicated treatment. After washing with PBS, the clones were fixed with methanol for 20 min and stained with 0.1% crystal violet solution, then washed with ddH_2_O several times and photographed.

### Flow cytometric analysis

The early apoptosis rates in 7703 and HepG2 cells under the indicated treatments were stained with Annexin-V/7-AAD according to manufacturer’s instructions and then subjected to flow cytometry.

### Tumorigenicity experiments

Cells were suspended in Matrigel/PBS (1:1) at a concentration of 5 × 10^7^ cells/ml. The cell mixture was injected subcutaneously into the left/right back of the mice (0.1 ml/per mouse). After 4 weeks, all mice were sacrificed, and the tumors were dissected. Tumor size was measured and calculated using the equation (length × width^2^)/2.

### Immunofluorescence

Cells were cultured for 24 h on culture slides and washed in PBS after adhering to slides. Then, the fixed cells were treated with 10% Goat serum albumin and incubated with primary antibody overnight. Following washing, secondary antibody was added, the slides were stained in DAPI (Vector Laboratories, CA, USA) and examined by confocal microscope.

### Western blot analysis

Tumor cells were lysed in the lysis buffer with a protease and phosphatase inhibitor cocktail. Western blotting was performed as standard protocol. Immobilon Western Chemiluminescent HRP Substrate (WBKLS0500, Millipore) was used for visualization and the signal was analyzed with Alpha Ease FC software (Bio-Rad, Hercules, CA).

### Co-immunoprecipitation (Co-IP)

Cells were treated with IP lysis buffer, then lysates were probed with antibody and magnetic beads at 4 °C all night. After that, samples were washed in PBS and eluted for western blot.

### Immunohistochemistry (IHC) analysis and TUNEL staining

Immunohistochemical analysis was performed as described previously [47]. In brief, tumor tissues were fixed in 4% paraformaldehyde and embedded in paraffin. Sections (4 μm) were prepared for indicated primary antibodies or hematoxylin and eosin (H&E) staining according to standard protocols. TUNEL Apoptosis Assay Kit was applied to validate the apoptotic cells according to manufacturers’ instructions (C1091, Beyotime, China).

### Cytosolic Ca^2+^ measurements

The intracellular Ca^2+^ concentration was calculated using Fura-4 Ca^2+^ Imaging Calibration kit (Invitrogen) according to the manufacturer’s protocol and equation.

### RT-PCR and quantitative real-time PCR (qRT-PCR)

Total RNA was extracted using TRIZOL Reagent (#15596-018, Invitrogen) and was reversed transcribed using cDNA reverse transcription kit (#RR037A, Takara). According to the manufacturer’s instructions, qRT-PCR with SYBR Green Master Mix (Vazyme) on StepOnePlus real-time PCR system (ABI). The primers used were shown in Table S2.

### RNA immunoprecipitation (RIP) assay

After culturing in RIP lysis buffer, lysates from processed cells were conjugated with antibody or control IgG antibody in magnetic beads. After RNA enrichment, qRT-PCR was implemented to analyze RNA enrichment. The primers used were listed in Table S3.

### Statistical analysis

All data were presented as mean ± SD. Student’s *t*-test (two groups) or one-way ANOVA (multiple groups) were applied to analyze the differences between groups by using SPSS 22.0. *P* values less than 0.05 were considered statistically significant.

## Supplementary information

Supplementary figure legends

supFig 1

supFig 2

supFig 3

supFig 4

Table S1

Table S2

Table S3

## Data Availability

The datasets used and/or analyzed during the current study are available from the corresponding author on reasonable request.
