# Correction to: CHD1L prevents lipopolysaccharide-induced hepatocellular carcinomar cell death by activating hnRNP A2/B1-nmMYLK axis

**DOI:** 10.1038/s41419-022-04648-5

**Published:** 2022-03-02

**Authors:** Guangliang Wang, Xiaofeng Zhang, Wei Cheng, Yanxuan Mo, Juan Chen, Zhiming Cao, Xiaogang Chen, Huiqin Cui, Shanshan Liu, Li Huang, Ming Liu, Lei Ma, Ning-Fang Ma

**Affiliations:** 1 Affiliated Cancer Hospital and Institute, Guangzhou Medical University, Guangzhou, China; 2 Department of Histology and Embryology, Faculty of Basic Medical Sciences, Guilin Medical University, Guilin, Guangxi China; 3 Guangzhou Municipal and Guangdong Provincial Key Laboratory of Protein Modification and Degradation, School of Basic Medical Sciences, Guangzhou Medical University, Guangzhou, China

**Keywords:** Cancer microenvironment, Apoptosis, Liver cancer

Correction to: *Cell Death and Disease* 10.1038/s41419-021-04167-9, published online 29 September 2021

The original version of this article, unfortunately, contained a mistake in the affiliations. The original article has been corrected.

