# Supplementary material for: CHD1L prevents lipopolysaccharide-induced hepatocellular carcinomar cell death by activating hnRNP A2/B1-nmMYLK axis
Source: Cell Death Dis. 2021 Sep 29;12(10):891. doi: 10.1038/s41419-021-04167-9 (PMC8481269; doi:10.1038/s41419-021-04167-9)
Supplement: Supplementary file 1 — Supplementary figure legends [file 41419_2021_4167_MOESM1_ESM.docx]

**Supplementary figures**

**Fig. S1. The expression of CHD1L and MYLK in HCC cell lines.** **A, B** Differential expression of CHD1L and MYLK in HCC cell lines were examined using qRT-PCR (**A**) and Western blot (**B**), with GAPDH as normalized control. Data represent mean ± SD with three independent experiments. ***P* < 0.01.

**Fig. S2. Downregulation of nmMYLK in HCC cells inhibits Cyclin D1 expression.** **A, B** The protein levels of Cyclin D1 regulated by nmMYLK in HepG2 (**A**) and 7703 (**B**) cells were examined using Western blot, with indicated treatment.

**Fig. S3. Downregulation of nmMYLK in 7703-CHD1L xenograft tumors promotes cascade reaction of caspases.** 7703-CHD1L cells infected with shnmMYLK lentiviruses were inoculated subcutaneously into nude mice, tumor tissue lysates were subjected to immunoblotting with the indicated antibodies. Data were performed by mean with SD from three independent experiments. ***P* < 0.01.

**Fig. S4. LPS induces Ca^2+^ entry and activates nmMYLK. A** Intracellular Ca^2+^ concentration was examined using Fura 4-AM in HepG2 cells after LPS treatment. **B** Expression of MLC2 and its phosphorylated were examined using Western blot in HepG2 cells after LPS treatment. Experiments were repeated for three times, data were shown as mean ± SD, ***P* < 0.01.
