# Supplementary figures and images for: CHD1L prevents lipopolysaccharide-induced hepatocellular carcinomar cell death by activating hnRNP A2/B1-nmMYLK axis
Source: Cell Death Dis. 2021 Sep 29;12(10):891. doi: 10.1038/s41419-021-04167-9 (PMC8481269; doi:10.1038/s41419-021-04167-9)

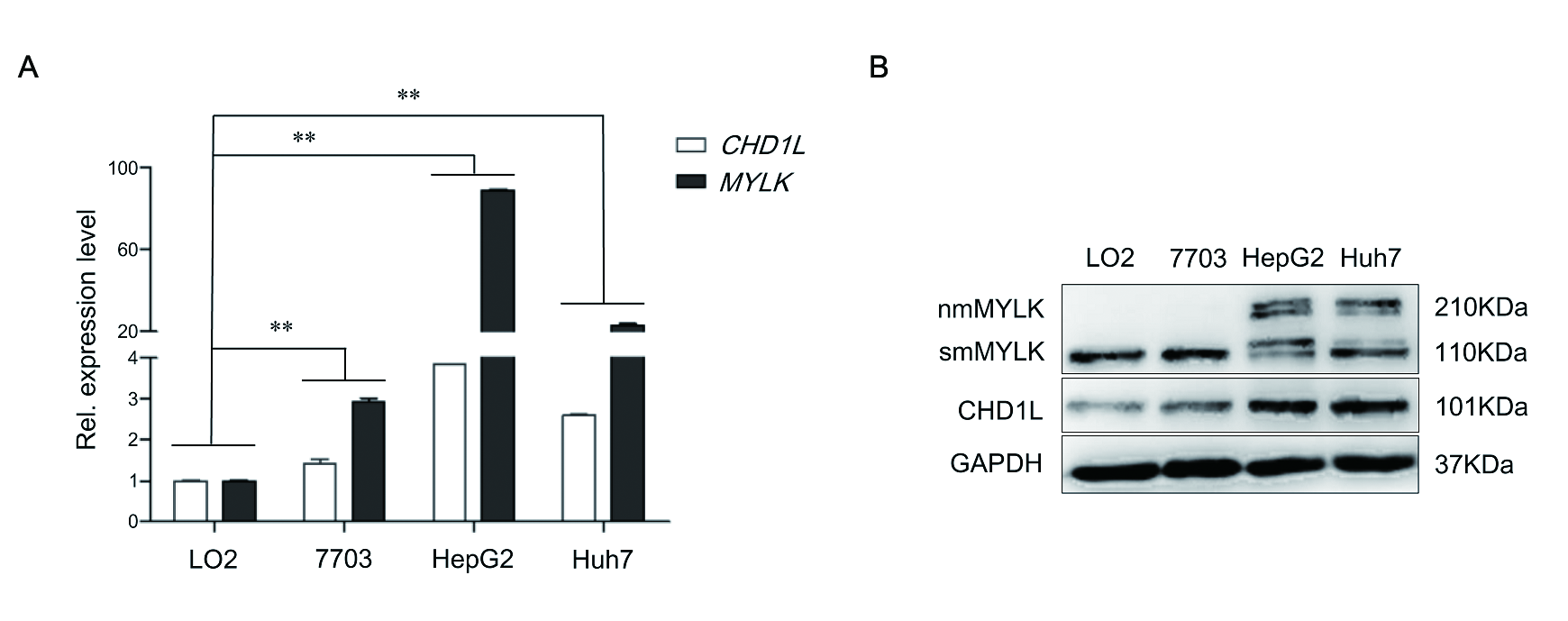

Supplement: Supplementary file 2 — supFig 1 [file 41419_2021_4167_MOESM2_ESM.tif]

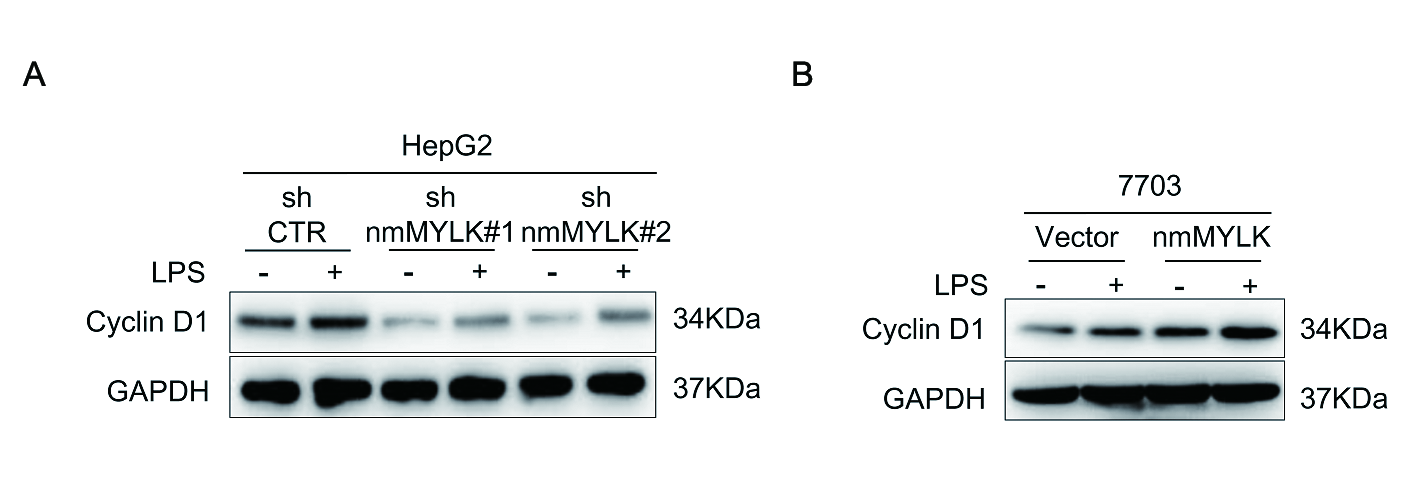

Supplement: Supplementary file 3 — supFig 2 [file 41419_2021_4167_MOESM3_ESM.tif]

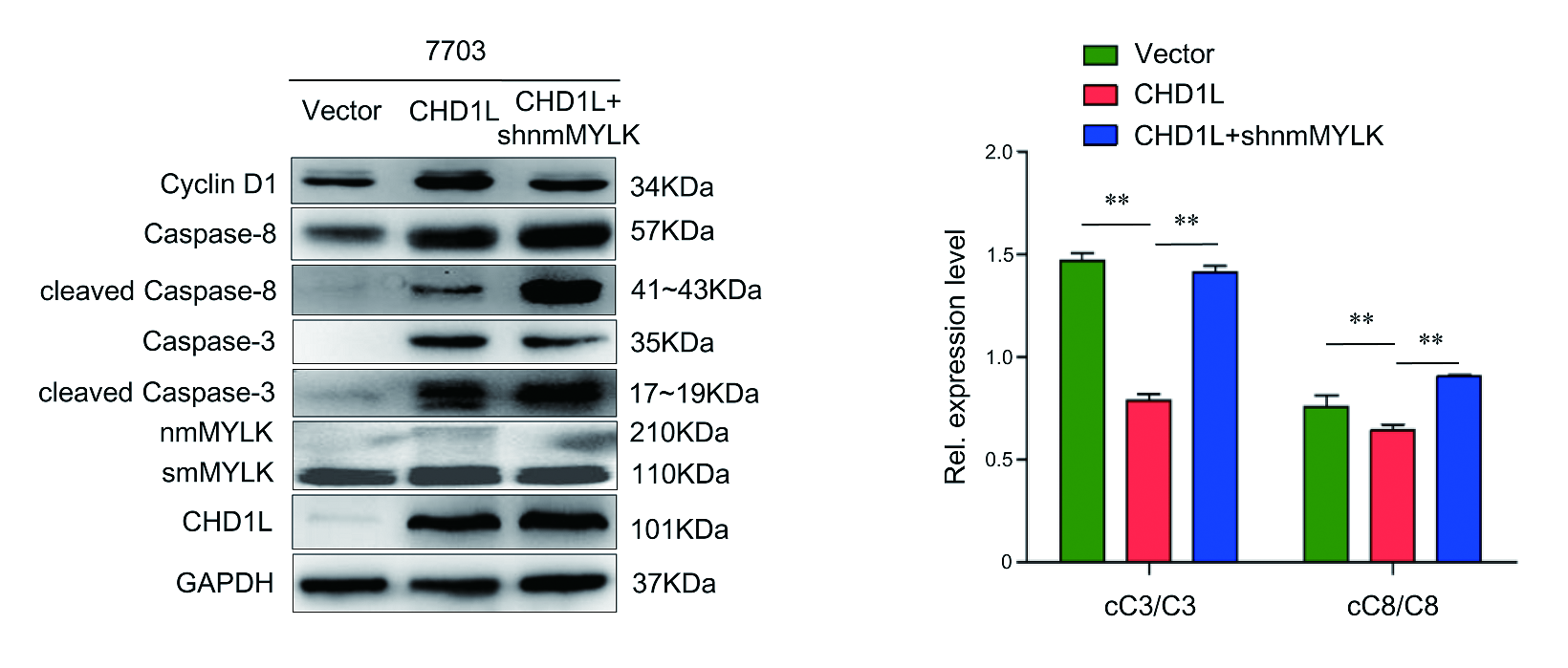

Supplement: Supplementary file 4 — supFig 3 [file 41419_2021_4167_MOESM4_ESM.tif]

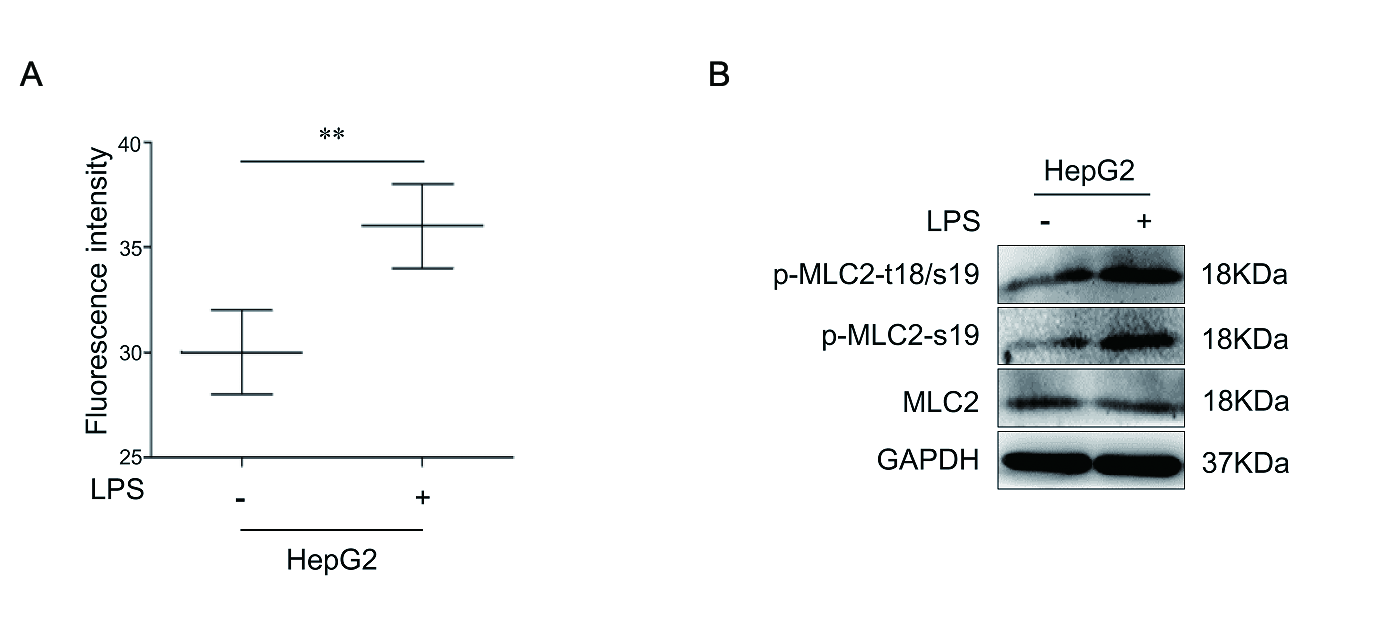

Supplement: Supplementary file 5 — supFig 4 [file 41419_2021_4167_MOESM5_ESM.tif]
