# Supplementary material for: CHD1L prevents lipopolysaccharide-induced hepatocellular carcinomar cell death by activating hnRNP A2/B1-nmMYLK axis
Source: Cell Death Dis. 2021 Sep 29;12(10):891. doi: 10.1038/s41419-021-04167-9 (PMC8481269; doi:10.1038/s41419-021-04167-9)
Supplement: Supplementary file 6 — Table S1 [file 41419_2021_4167_MOESM6_ESM.docx]

Table S1. Sequence information for siRNA and shRNA fragment used in described studies:

| Interference fragment | | Sequence |
| --- | --- | --- |
| *CHD1L* siRNA#1 | Sence 5'- CCUGCUGGAUAAGCUACUAdTdT -3' | |
| *CHD1L* siRNA#1 | Antisence 5'- UAGUAGCUUAUCCAGCAGGdTdT -3' | |
| *CHD1L* siRNA#2 | Sence 5'- CCAAACUGCAGCUCACCAAdTdT -3' | |
| *CHD1L* siRNA#2 | Antisence 5'- UUGGUGAGCUGCAGUUUGGdTdT -3' | |
| *nmMYLK* shRNA#1 | Sence 5'- GAUUUGACUGCAAGAUUGAdTdT -3' | |
| *nmMYLK* shRNA#1 | Antisence 5'- UCAAUCUUGCAGUCAAAUCdTdT -3' | |
| *nmMYLK* shRNA#2 | Sence 5'- GCCACUUCCAGAUAGACUAdTdT -3' | |
| *nmMYLK* shRNA#2 | Antisence 5'- UAGUCUAUCUGGAAGUGGCdTdT -3' | |
| *hnRNP A2/B1* siRNA#1 | Sence 5'- GCUGUUUGUUGGCGGAAUUdTdT -3' | |
| *hnRNP A2/B1* siRNA#1 | Antisence 5'- AAUUCCGCCAACAAACAGCdTdT -3' | |
| *hnRNP A2/B1* siRNA#2 | Sence 5'- GGAGAGUAGUUGAGCCAAAdTdT -3' | |
| *hnRNP A2/B1* siRNA#2 | Antisence 5'- UUUGGCUCAACUACUCUCCdTdT -3' | |
