# Supplementary material for: CHD1L prevents lipopolysaccharide-induced hepatocellular carcinomar cell death by activating hnRNP A2/B1-nmMYLK axis
Source: Cell Death Dis. 2021 Sep 29;12(10):891. doi: 10.1038/s41419-021-04167-9 (PMC8481269; doi:10.1038/s41419-021-04167-9)
Supplement: Supplementary file 7 — Table S2 [file 41419_2021_4167_MOESM7_ESM.docx]

Table S2. Sequence information for primers used in qRT-PCR

| Gene | Forward sequence | Reverse sequence |
| --- | --- | --- |
| *18S* | 5'-AACCCGTTGAACCCCATT -3' | 5'-CCATCCAATCGGTAGTAGCG -3' |
| *CHD1L* | 5'- GCTTCTTACTGCGGCTTCATACT-3' | 5'-TGCTCAAAACAGACAAGGGACA-3' |
| *MYLK* | 5'-TTCCGGGATTCCAAAGCCTG-3' | 5'-GTGCAGCTGTATGTCCCACT-3' |
| *hnRNP A2/B1* | 5'-TGTTCCTTTGGAGAGGAAAAAGAGA-3' | 5'-TGCTTGCAGGATCCCTCATT-3' |
| *EHD1* | 5'-GACAACAAGCCTATGGTGCTC-3' | 5'-AAGTCCTGCTCGATCAGGTGT-3' |
| *NFATC1* | 5'-CACCGCATCACAGGGAAGAC-3' | 5'-GCACAGTCAATGACGGCTC-3' |
| *SDC4* | 5'-GGACCTCCTAGAAGGCCGATA-3' | 5'-AGGGCCGATCATGGAGTCTT-3' |
| *CyclinD1* | 5'-TGGATGCTGGAGGTCTGCGA-3' | 5'-CGGATGGAGTTGTCGGTGTAG-3' |
| *cIAP1* | 5'-CCAGCCTTTCTCCAAACCCTC-3' | 5'-ACAGGCAAAGCAGGCTACCC-3' |
| *ICAM-1* | 5'-ACCTTACCCTACGCTGCCAG-3' | 5'-AGGTCCAGTTCAGTGCGGCA-3' |
