# Supplementary material for: CHD1L prevents lipopolysaccharide-induced hepatocellular carcinomar cell death by activating hnRNP A2/B1-nmMYLK axis
Source: Cell Death Dis. 2021 Sep 29;12(10):891. doi: 10.1038/s41419-021-04167-9 (PMC8481269; doi:10.1038/s41419-021-04167-9)
Supplement: Supplementary file 8 — Table S3 [file 41419_2021_4167_MOESM8_ESM.docx]

Table S3. Sequence information for primers used in RIP assay

| Gene | Forward sequence | Reverse sequence |
| --- | --- | --- |
| *U1* | 5'-GGGAGATACCATGATCACGAAGGT -3' | 5'-CCACAAATTATGCAGTCGAGTTTCCC-3' |
| *MYLK#1* | 5'-TGAGCCACTTTATTCCAAAGTAC-3' | 5'- CCAGAGTCATCCATGAGCAG-3' |
| *MYLK*#2 | 5'-CATGCTGTCCATGAGGAG-3' | 5'- CTCCACTGCTGGAGCTG-3' |
